# ‘Differences between the earth and the sky’: migrant parents’ experiences of child health services for pre-school children in the UK

**DOI:** 10.1017/S1463423620000213

**Published:** 2020-08-17

**Authors:** Louise Condon, Stuart McClean, Luiza McRae

**Affiliations:** 1 Department of Nursing, Swansea University, Swansea; 2 Department of Health and Social Sciences, University of the West of England, Bristol; 3 Freelance Interpreter

**Keywords:** child health, child health promotion, migrant health, primary care, qualitative methods, Roma, surveillance

## Abstract

**Aim:**

To explore parents’ experiences of using child health services for their pre-school children post-migration.

**Background:**

Migrating between countries necessitates movement and adjustment between systems of healthcare. Children of migrants are known to have poorer health than local children on some measures and are less likely to access primary care. In the United Kingdom (UK), children are offered a preventive Healthy Child programme in addition to reactive services; this programme consists of health reviews and immunisations with some contacts delivered in the home by public health nurses.

**Methods:**

Five focus groups were held in a city in South West England. Participants were parents of pre-school children (n = 28) who had migrated to the UK from Romania, Poland, Pakistan or Somalia within the last 10 years. Groups selected included both ‘new migrants’ (from countries which acceded to the European Union in the 2000s) and those from communities long-established in the UK (Somali and Pakistani). One focus group consisted of parents of Roma ethnicity. Interpreters co-facilitated focus groups.

**Findings:**

Participants described profound differences between child health services in the UK and in their country of origin, with the extent of difference varying according to nationality and ethnic group. All appreciated services free at the point of delivery and an equitable service offered to all children. Primary care services such as treatment of minor illness and immunisation were familiar, but most parents expected doctors rather than nurses to deliver these. Proactive child health promotion was unfamiliar, and some perceived this service as intruding on parental autonomy. Migrants are not a homogenous group, but there are commonalities in migrant parents’ experiences of UK child health services. When adjusting to a new healthcare system, migrants negotiate differences in service provision and also a changing relationship between family and state.

## Background

Migration of people between countries has been growing exponentially since the late 20th century (International Organisation for Migration, 2013), with the countries of departure varying according to world events and changing motivational or deterrent factors (McDowell, 2008). Migration from Eastern Europe has increased since the accession to the European Union of Poland in 2004 and Romania in 2007, which gave their citizens the same rights to work as UK citizens (Rutter, 2015). A migrant is defined as a person born abroad who intends to stay in the country of settlement for at least one year (United Nations Statistical Commission, 1998; Markkula *et al*., 2018), and the majority of migrants move to a country in which they are entitled to live and work (Rutter, 2015). Contrary to popular belief, a very small percentage of the UK population are refugees (currently estimated at 1%) (Refugee Council, 2019). Migrants are most commonly young adults who bring children with them or start a family once settled in their new country.

The health of migrants is a topic of policy interest although there is a lack of objective data. UK data sets frequently do not make distinction between ethnicity and country of origin, which obscures migration history (Jayaweera, 2014). Migrants have varying degrees of financial, social and cultural capital (Jayaweera and Quigley, 2010) which affects their socioeconomic status post-migration and hence health outcomes. However, migrants in Europe have poorer self-reported health than the majority population (Nielsen and Krasnik, 2010; Jayaweera, 2014), and many experience high social deprivation (Tulchinsky and Varavikova, 2014).

Research indicates that children of migrants to Europe have specific health needs, including higher rates of obesity, dental caries and some infectious diseases (Labree *et al*., 2011; Jaeger *et al*., 2012; Gualdi-Russo *et al*., 2014). A recent systematic review reported that first and second generation international migrant children (0–18 years) use most types of healthcare services less than local children, with only emergency and hospital services used more (Markkula *et al*., 2018). Barriers to healthcare use among European migrants comprise language barriers, fear of stigma and lack of trust, financial difficulties and problems in navigating a new healthcare system (Simon *et al*., 2015). Difficulties in registering with a general practitioner (GP) pose a barrier to both primary care and child health promotion (Gazard *et al*., 2015; Condon and Mytton, 2019).

Experience of healthcare in the country of outward migration influences migrants’ expectations of the UK National Health Service (NHS) (Sime, 2014). Many European countries have insurance-based healthcare systems, and in all countries, private healthcare may co-exist with models of public provision. The UK has a government-funded system of universal healthcare with well-developed primary care, which differs from countries such as Romania and Poland where primary care is the focus of development (Vlãdescu *et al*., 2016; World Health Organization, 2018; 2019). In the UK, GPs act as a gateway to other NHS services through a process of referral, a system that may be unfamiliar to migrants (Hargreaves *et al*., 2006). In the UK, preventive healthcare for children is delivered via the Healthy Child Programme of each constituent country and includes immunisation and developmental review, plus child health promotion directed at parents and carers (Burton, 2019). This public health service predates the establishment of the NHS and is led by health visitors (community public health nurses) who have an established entrée into the home from pregnancy to school entrance (Peckover, 2002). By contrast, in countries with private medical services, doctors commonly review child development (Wilson and Law, 2019).

This paper reports findings from a range of migrant parents on their experiences of using primary care and health promotion services for pre-school children. It is part of a larger study that explored how migrant parents keep children healthy post-migration; findings on health behaviours have been reported elsewhere (Condon and McClean, 2016). Use of health services is a key part of maintaining child health in the early years but service providers suggest that primary care and universal health promotion are often unfamiliar to migrant parents (Gill, 2009). The early years are increasingly recognised as the foundation of physical and mental health through the life course (Hughes *et al*., 2017).

## Methods

### Design

An exploratory qualitative study design was used to give voice to participants, including those with no or limited English. The focus group method was selected to reduce the imbalance of power between researchers and participants who may be subject to impoverishment, discrimination and stigma and hence vulnerable; in a group discussion, individuals have more ability to express opinions, challenging the ‘authoritative voice’ and control of researchers (Liamputtong, 2007). To facilitate authentic participation, all groups were offered the opportunity to speak in their first language, apart from the Roma group where no Romanes interpreter existed. Roma participants were included because Roma people are subject to extreme social exclusion and socioeconomic deprivation across Europe and have poorer health and access to healthcare (Parekh and Rose, 2011; Cook *et al*., 2013; European Agency for Fundamental Rights, 2014). Public involvement was conducted with community members (linkworkers) who had experience of acting as interpreters and advocates to their respective communities in health settings. Linkworkers had themselves migrated to the UK and were therefore able to offer insights on study design and conduct from an insider perspective. One linkworker (LM) was a member of the research team.

### Ethics

Ethical approval was obtained from a University Ethics Committee. Written information about the study was circulated (in English or translation) to potential participants one week before the focus group. Written informed consent was obtained from all participants immediately prior to each focus group. After the group discussion, each participant was offered a supermarket voucher as a ‘thank you’ for their time.

### Participants and procedure

Inclusion criteria were to be a parent of a pre-school child and to have migrated to the UK in the last 10 years from Romania, Poland, Pakistan or Somalia. The sample was selected purposively to include established and recent migrant communities and different experiences of healthcare. Table 1 gives brief information on included groups and the health systems and economies of the countries of their outward migration.

**Table 1.** Details of selected migrant communities in the United Kingdom (UK) and the health systems of their country of origin

Country of birth	Estimate of population size in UK^1^	Notes on migration routes into UK^2^	Gross domestic product (GDP) of country of birth ($m)^3^ (% of GDP spent on healthcare)^4^	Health system in country of birth
Poland	679,000	Recent European Union (EU) migrants with some Second World War arrivals and post-war refugees	585.8 (6.3%)	Mandatory health insurance, complemented with financing from state and territorial self-government budgets. The National Health Fund manages contracts with public and non-public healthcare providers. Health insurance contributions are borne entirely by employees.^5^
Pakistan	502,000	Long-settled post-1950 migration, family migrants and recent student migrants	312.6 (2.7%)	Urban/rural disparities in healthcare delivery and an imbalance in the health workforce, with insufficient workers in the peripheral areas. Complex system where healthcare subsystems compete with formal and informal private-sector healthcare systems.^6^
Romania	130,000	Recent EU migrants	239.6 (5%)	Social health insurance system developed from the Semashko system which aimed for universal basic healthcare. Comprehensive benefits package to those insured and minimum benefits package if not covered. Inequities in healthcare access, for example, urban/rural.^7^
Somalia	97,000	Asylum and EU onward migration	7.5 (no data)	Somalia’s public healthcare system was largely destroyed during the civil war. The healthcare system in Somalia remains weak, poorly resourced and inequitably distributed. Health expenditure remains very low and there is a critical shortage of health workers.^8^
UK	66 million (total UK population)	Not applicable for total population	2,825 (9.9%)	Government funded system of universal healthcare with well-developed primary care and an established child health promotion programme.

1 ONS (2018) Population of the UK by country of birth and nationality: individual country data. https://www.ons.gov.uk/

2 Adapted from Rutter (2015).

3 World Bank-Poland https://data.worldbank.org/indicator/NY.GDP.MKTP.CD?locations=PL ;Pakistanhttps://data.worldbank.org/indicator/NY.GDP.MKTP.CD?locations=PK; Romania https://data.worldbank.org/indicator/NY.GDP.MKTP.CD?locations=RO; Somalia - https://data.worldbank.org/indicator/NY.GDP.MKTP.CD?locations=SO

4 WHO (2019) Current health expenditure as a percentage of gross domestic product (GDP) https://www.who.int/gho/health_financing/health_expenditure/en/

5 WHO Health system review. Health Systems in Transition, 2011, 13(8):1–193. http://www.euro.who.int/__data/assets/pdf_file/0018/163053/e96443.pdf

6 WHO Country assessment: http://www.emro.who.int/pak/programmes/service-delivery.html

7 WHO Europe – Romania: Regions for Health Network http://www.euro.who.int/en/about-us/networks/regions-for-health-network-rhn/activities/regional-profiles/romania/romania

8 WHO Humanitarian Response Plan 2015 https://www.who.int/hac/donorinfo/somalia.pdf

Recruitment was carried out by the four linkworkers from Pakistani, Polish, Somali and Romanian backgrounds who acted as gatekeepers to their communities. Each linkworker identified up to eight participants and invited them to attend at an agreed date and time. LM acted as linkworker for two groups (Romanian and Roma) and was a member of the research team; though not of Roma ethnicity, LM has extensive experience of working with this community.

Five focus groups were held with 28 parents (see Table 2 for demographic details). Both fathers and mothers were invited, but just six fathers participated in focus groups, all of Eastern European nationality.

**Table 2.** Demographic details of participants (*n* = 28)

	Sex	Age	Highest educational qualification	Employment	Number of children	Length of residency in UK
**Romanian (** ***n*** **= 7)**	5 × mothers 2 × fathers	Range 18–35 years (mean 30 years)	7 × A level equivalent	1 × employed 1 × student 1 × seeking work 4 × not known	Range 1–2 children (mean1)	Range ≤1–4 years (mean 2 years)
**Roma (** ***n*** **= 6)**	4 × mothers 2 × fathers	Range 17–47 years (mean 30 years)	6 × no qualifications	2 × employed 1 × seeking work 3 × mothers with no paid work	Range 1–8 children (mean 3)	Range ≤1–5 years (mean 3 years)
**Polish (** ***n*** **= 6)**	4 × mothers 2 × fathers	Range 28–36 years (mean 33 years)	2 × Master’s degrees 2 × Bachelor’s degrees 2 × A level equivalent	4 × employed 1 × student 1 × seeking work 1 × mother with no paid work	Range 1–2 children (mean 1)	Range 2–9 years (mean 7 years)
**Somali (** ***n*** **= 5)**	5 × mothers	Range 27–43 years (mean 35 years)	2 × A level equivalent 3 × vocational qualifications	2 × employed 3 × mothers with no paid work	Range 1–4 children (mean 2)	Range 5–9 years (mean 6 years)
**Pakistani (** ***n*** **= 4)**	4 × mothers	Range 29–35 years (mean 31 years)	2 × A level equivalent 2 × GCSE equivalent	4 × mothers with no paid work	Range 2–4 children (mean 2)	Range 1–10 years (mean 5 years)

### Data collection and analysis

Focus groups took place in community venues between January and March 2015 in a city in South West England. All focus groups were led by LC or SM and co-facilitated by a linkworker who provided concurrent translation, meaning that the data were recorded in English. The Polish group was conducted in English at the request of participants. Questions relating to use of health services were included in a topic guide which was designed to explore parental health behaviours post-migration. Box 1 shows the questions and prompts that relate specifically to health service use. Focus group discussions lasted 60–90 minutes.

Box 1. Questions and prompts concerning health service use for pre-school children

Topic Guide

1. Tell me about being a parent in the UK, is it different from your home country?

Prompt: Healthcare for pre-school children

1. Do you think moving to the UK has influenced your child’s health at all?

Prompts: Use of health services (ease of access, attitudes encountered, satisfaction with service offered); accidents, immunisations.

NVivo10 was used to store and categorise data. A thematic content analysis approach was taken which offers a flexible approach to analysing qualitative data (Braun and Clarke, 2006; Vaismoradi, Turunen and Bondas, 2013). Initially data were coded to identify preliminary themes by LC and SM, and immersion in the data led to the inductive development of ideas and broader observations, which were discussed by the research team (Bowling, 1997). Data from the five nationalities/ethnicities were compared and contrasted in terms of emergent themes.

## Results

Results are organised into four overall themes developed from the analysis. These are Comparison between Health Services; A Child’s Right to Healthcare; Consumer Choice and Health Promotion. Cross-cutting themes are parental autonomy and governmentality, which are interwoven within the narrative account below and later discussed. Governmentality is the term used by Foucault (1975) to describe the ways in which social life is directed and influenced by the state; this is done by establishing societal norms (how people should think and behave) and is encouraged by expert-based knowledge. Parenting is a well-discussed site of governmentality (Rose, 1990; 1993; Dahlstedt 2009). Direct quotations are presented anonymously with each participant’s gender, nationality or ethnicity, and age given.

### Comparison between health services

All migrant groups described major differences between health systems in their own countries and the UK, summed up as ‘differences from earth to the sky’ (Roma mother, 34 years). Basic components of the healthcare system were similar (e.g. immunisations), but differences arose in the personnel offering these services, how services were accessed and paid for and the perceived quality of the services provided. Participants in all groups showed a good understanding of the child health services available post-migration and most expressed satisfaction with the ease of access. A Somali mother listed the services she would use:

*If your child not well you can either phone, take to the emergency if health centre not open, you can book an appointment to see one of the doctors, if…you are not sure about something you can talk to your health visitor. It’s easy, no problem.* Somali mother, 38 years

Participants identified the GP as the usual entry point to the healthcare system and the source of referrals to secondary care. Satisfaction with UK services was highly dependent upon healthcare experiences in the country of origin. While appreciating state provision of health services, those who previously enjoyed access to private medicine found it difficult to adapt to the lack of choice within the NHS. Parents from Pakistan, Somalia and Romania described easy access to private health services in their country of origin, which offered quicker access to assessment and treatment if service users could afford to pay. If you paid for healthcare, you could consult the professional of your choice; this was important for Polish participants who were concerned about the ability of nurses to assess and treat ill children. Romanian parents described how waiting time was reduced and test results received sooner in a private system.

Stark contrasts were apparent in the accounts of health systems given by Roma and Romanians, despite both originating from Romania. Romanian parents described an accessible insurance-based system, with a parallel private healthcare system [‘We do prefer to go to the private hospital if you have money’ (Romanian mother, 30 years)]. By contrast, Roma parents described hospitals as being positively injurious to health [‘they are not clean, there are rats, cockroaches…bacteria, infections’ (Roma mother, 34 years)]. Some Roma parents described having to bribe hospital personnel in order to obtain services, even for a child:

[Here] *you don’t have to give them money or presents like it is with us, in Romania you give bribery, in the hospitals everywhere, they don’t touch the person or the child, the child can die unless you give him money, unfortunately this is how it is*. (Roma mother, 17 years)

Most groups found little difference in immunisation procedures; however, one Somali mother interpreted reminders to immunise as pressure to comply:

*Immunisation here like, for example you get reminders, you have to immunise your children, but back home you have a choice; you can take only if you want, nobody would push you to do that, so it’s just like, take or not take.* Somali mother, 43 years

Parental freedom to choose in Somalia was compared with monitoring of uptake in the UK, which rendered immunisation as compulsory in this mother’s eyes. Resistance to governmentality (here the organisational normalisation of childhood immunisation) is apparent in her response.

### A Child’s Right to Healthcare

Services free at point of delivery were appreciated by all parents, and they valued their child’s right to healthcare in the UK. Most participants considered that they benefited from this policy of equal access, and the contrast with systems in some countries of departure was implicit:

*Here every child gets treated the same, there’s no like, you’re rich, you’re poor, and you’re going to get the treatment…back home is the difference you know, if you’re rich your child gets five star service.* Pakistani mother, 29 years

Pakistani, Somali and Roma parents all described children as at risk of dying in their countries of origin if their parents could not pay for medical care. For the Roma, who described severe hardship in their country of origin, the level of inbuilt statutory equality in the UK seemed remarkable. Even where health services were considered basically comparable, some aspects were noted as preferable in the UK, for instance, less queuing at the doctor’s surgery, and children’s glasses being on prescription (Polish group). All groups appreciated free prescriptions for children’s medications, especially when parents had to juggle childcare with paid work:

*The medicine when your child is ill is free in the UK, whereas in Poland and your child is ill and you go to the doctor you are coming back with a list of medication you need to buy which is extremely expensive and you need to pay for it… you have to stay at home because you’re not going to work, you’re not going to be paid, but you still have to buy medicines.* Polish mother, 28 years

Three instances were given of poor service for children in the UK. Firstly, Polish and Romanian parents perceived doctors in their own countries as more thorough, careful and intent on giving a good patient-oriented service. Polish parents resented being asked if the ‘child was OK’ prior to an immunisation given by a nurse, where their normative assumption was that the doctor would review the child’s health and make the decision to immunise or not. Similarly, when an ill child was brought to the doctor, the expectation was to have a full medical examination:

*In Romania, at home if you go with a child at the doctor they check him from head to toe…and they check their reflexes… weight…everything but here we’ve been with temperature, and they just gave him some Nurofen, paracetamol.* Romanian mother, 34 years

Secondly, some Romanian parents were distrustful of medication prescribed in the UK for childhood ailments (such as cough or nappy rash), considering that remedies available in Romanian pharmacies were more effective. Parents therefore purchased these on trips abroad. Conversely, Somali mothers spoke of herbal remedies which their mothers continued to recommend, but stated that they would not use these when they could access UK medical care.

Finally, a Romanian father was not able to register his baby son at a GP practice until he was four months old, leading to delayed immunisation.

*We found it difficult to register our boy to a GP in Scotland…we tried online, and I had to take a lot of time off from work to be able finally to do it…*[This was] *because I wasn’t noticed, I wasn’t given importance.* Romanian father, 28 years

The father attributed this to insufficient attention being paid to him by staff at the GP practice. Discrimination was also discussed in the Roma group (where it was perceived as a fact of life and present in all societies), but no further examples of discrimination were given by any group.

### Consumer choice

Consumer choice was most apparent in relation to medications for children, particularly prescribing of antibiotics. There was a view in all groups that medication in the NHS was grudgingly given and that the most simple and least potent of remedies were suggested. One parent commented: ‘Here is the kingdom of paracetamol*’* (Roma father, 47 years). Pre-migration medication was described as more widely prescribed with Pakistani mothers describing children’s diarrhoea and vomiting commonly being treated by an intravenous drip, accompanied by ‘four to five*’* medications. The Romanian group also contrasted doctors ‘back home’ freely giving ‘lots of pills’ to children, to the UK where parents need to ‘beg’ for medication.

In a further extension of parental autonomy, Pakistani and Somali mothers described being able to access antibiotics from pharmacies in their home countries if necessary. Roma parents disputed the availability of antibiotics in Romania; some maintained that it was only possible to access antibiotics via a doctors’ prescription, while others claimed bribery could be used to access the drugs one wanted. In all groups, there were examples of changing attitudes to antibiotics, with parents increasingly adopting the UK stance that relatively few ailments require antibiotic treatment. One Polish father, who had lived in the UK for seven years, said:

*Actually when I came here and when* [child] *was young I thought that the Polish had done it better because they gave you antibiotics and that’s good, and here they give you paracetamol, ibuprofen, but now I think that is better*. Polish father, 35 years

This developing knowledge in the light of new social norms is indicative of progressive acceptance of governmentality in relation to antibiotic use.

Awareness was shown of health promotion messages about antibiotics (‘Even in Poland authorities say antibiotics will not kill viruses’, Polish father, 33 years), but parents considered that within the Polish system, doctors were reluctant to risk the clinician/patient relationship by denying antibiotics. In the UK, Pakistani mothers were confident that if antibiotics were refused on a first visit, a child could be taken back if necessary for reassessment. They showed general trust in the UK health system and belief that, while little medical care would be lavished on the child with a minor ailment, a seriously unwell child would receive appropriate treatment.

### Health promotion

Preventive aspects of the UK health system were new to all groups, including routine Healthy Child programme contacts. Parents were aware of the emphasis in the UK on ‘healthy living’ as a means of preventing ill health, including the promotion of healthy diet, exercise and reducing the risk of accidents. For some Polish parents, this governmental expectation of citizens to optimise their health status, irrespective of the constraints of their socio-economic circumstances, was an unfamiliar concept:

[In Poland] *they don’t have wellness, they don’t have healthy living. Healthy lifestyle, they don’t have the time, they don’t have the money, they are average people*. (Polish father, 33 years)

Living in a society which valorises children’s well-being and safety brought new responsibilities for parents as well as many benefits for children. These responsibilities needed to be fulfilled without support from the extended family. Family and community support was described by Somali, Pakistani and Romanian parents as the norm in their own countries. UK Healthy Child Programmes prescribe some visits to the family at home. A Somali mother commented:

*The midwife come and see you and the health visitors come and see you, and then they keep on seeing you, and then if the child has any problem they keep coming and guiding us*. Somali mother, 34 years

Their services reached right into the heart of family life, even changing the food offered to children. Demonstrating governmentality through expert knowledge, Pakistani mothers described being ‘trained’ to offer boiled, mashed vegetables as a weaning food in place of the customary chapatti, followed by curry when the baby was older. One mother drew a distinction between services ‘back home’ where a mother could choose to consult a practitioner about immunisation at the hospital, with unsolicited visits from midwives and health visitors. Such unsolicited visits would not happen in Pakistan because ‘when you go home with the baby nobody comes’ (Pakistani mother, 30 years).

There was no sense in any group that preventive services were refused or resented; however, they were seen as a part of a system that involves monitoring and assessment of parenting. Amusement was expressed in the Somali group about health visitors giving advice about feeding and safety but no practical help (‘They give enough advice…but…they’re not going to do the housework’, Somali mother, 27 years). Pakistani, Somali and Polish parents described safety advice being given in health and education settings and perceived a pervasive concern about risk to children within UK society. Somali and Polish parents considered potential risks to children were over-emphasised, agreeing that this increased parental anxiety (‘I think you got to be a bit careful because … we’re going to end up…seeing danger behind everything, everyone’. Polish mother, 28 years). Some described parental autonomy as greater pre-migration. A Pakistani mother explained the difference:

[There] *mum and child can do anything, if the child falls there’s nowhere to report. Here everybody is interested* [laughs] *you get the social worker involved, the hospital people and doctor involved; nobody has responsibility back home, only the mum*. (Pakistani mother, 35 years).

Thus, seeking help after an accident could result in the parent becoming the focus of professional concern. Parents perceived that using child health services was a two-way process with service providers sharing information with other agencies and assessing parenting. Fear was expressed in the Somali group that children could be removed from parents in the UK for child protection reasons.

## Discussion

Migration flows are characterised by more diversity and cultural complexity than ever before, leading to ‘superdiversity’ within society (Vertovec, 2008; Phillimore, 2016). Jayaweera (2014) has highlighted the heterogeneity of the UK migrant population, and Erel (2010) the impact of differing levels of social and cultural capital upon expectations of healthcare. Both these points are reflected within this study, which included participants diverse in nationality, ethnicity, educational attainment and access to healthcare pre-migration. Pre-migration experiences of healthcare are associated with ethnicity as well as nationality, as exemplified by the contrasting accounts of pre-migration healthcare given by Romanian and Romanian Roma parents. Notwithstanding these differences, this study suggests that post-migration there are commonalities in migrant parents’ experiences of the UK health system. Parents’ views of child health services are important to providers and commissioners of services as pre-school children rely completely on adults to gain access to appropriate healthcare.

The most familiar services were those sought when a child was unwell or injured. Unlike some previous research (Phillimore, 2016), all participants were aware of the role of the GP as the gateway to more specialised services. One Eastern European parent encountered difficulties in registering with a GP, a problem replicated in several UK studies of a variety of migrant groups (Hargreaves *et al*., 2006; Aung *et al*., 2010; Stagg *et al*., 2012; Sime, 2014; Gazard *et al*., 2015). Existing research also indicates that Eastern European adults and children may perceive UK doctors as less skilful and thorough than doctors in their country of origin (Sime, 2014; Bell *et al*., 2019). In this study, Polish and Romanian parents were familiar with more consumer-orientated medical services, either available through the state or privately. This was particularly apparent in relation to antibiotic prescription, where participants described more room for negotiation in their country of origin (Polish and Romanian parents). Sytnik-Czetwertynski and Cianciara (2016: 223) suggest that in an expert-led health system with a public health approach, there is little room for dialogue with the individual. This proved problematic for parents in this study who had previously exercised parental autonomy by using private healthcare or accessing the medications they wanted via pharmacies.

This study also displayed parents’ unfamiliarity with nurses working in advanced roles in primary care, which has been noted previously (Bell *et al*., 2019). Global and national policy promotes the skills and capabilities of nurses, and their role in enabling universal health coverage (World Health Organization, 2016; All-Party Parliamentary Group on Global Health, 2016), but this study suggests that communication of benefits to service users has lagged behind policy change. Developing greater trust in primary care practitioners is a key factor in optimising migrant children’s health service use (Bell *et al*., 2019), and particularly important in cross-cultural consultations with migrants (Gill, 2009; Muijsenbergh *et al*., 2014). It is likely that increasing confidence in all primary care services would contribute to reducing the use of Accident and Emergency departments, which reliably provide access to a doctor and physical examination, services that meet many migrant parents’ cultural expectations of healthcare and are highly valued. Raising awareness of the skills of nurses could be implemented at a policy level by a public information campaign, or in practice by primary care professionals and health visitors routinely giving clear explanations of specialist roles.

Common to all groups was unfamiliarity with child health promotion as a component of preventive healthcare. At its broadest level, this is an injunction to live healthily and for parents to ensure that children are raised in a healthy environment. Socialising individuals to take on state messages and enact them in the home is a central theme of Foucault’s concept of governmentality. Governmentality enables public surveillance and heightens personal responsibility as individuals and families are perceived to be in control of health and social risks (Foucault, 1975; 1991; Coveney, 1998; Ristovski-Slijepcevic *et al*., 2010). Such messages do not take into account the varying abilities of parents to prioritise their children’s health and ensure their well-being, irrespective of their living and working conditions. This conundrum is particularly acute for migrant parents who are likely to experience downward social mobility (McDowell, 2008) and be among the most disadvantaged within their new social strata (Davies *et al*., 2009). This poses challenges to those who promote child health, particularly health visitors who are the lead agency in delivering Healthy Child programmes. Health visiting’s connotations of governmentality have been well discussed (Peckover, 2002; Brownlie and Howson, 2006; Peckover and Aston, 2018) and merit further exploration in relation to superdiversity within 21st century society. To work well with migrant families, practitioners require sensitivity to cultural factors and parents’ understanding of health conditions, as well as the ability to develop trust over time (Condon and Mytton, 2019). This has implications for the training of primary care practitioners and health visitors, and for the commissioning and planning of continuity of care.

### Limitations and strengths of the study

A strength of this study was the inclusion of recent migrants from Eastern Europe and ex-Commonwealth and colonial communities, and fathers as well as mothers. Limitations include each national and ethnic group being small, and because participants were known to linkworkers, less likely to be socially excluded. Participants had moved to the UK in the last 10 years, but length of time since migration varied between groups. As is common with cross-cultural studies, researchers were limited by their inability to speak the languages of all participants. To an extent, this was ameliorated by LM, a Romanian speaker, translating for two groups; however, project funding did not allow audio recordings to be checked by an objective translator. The lack of a Romanes-speaking interpreter for the Roma group was also a limitation, as this may have constrained their ability to fully express themselves. Linkworkers were familiar to participants from other settings (health and education), which may have inhibited participants’ freedom to speak. Language difficulties were not raised here despite being reported elsewhere (Sime, 2014; Bell *et al*., 2019); this is a possible artefact of the dual role of linkworkers and interpreters in this study. Interpretation is accepted as a key factor in forging links between health workers and service users (Hadziabdic *et al*., 2009) and increasing trust between practitioners and service users (Muijsenbergh *et al*., 2014). Familiarity with some Roma participants from previous research may have contributed to the taboo subject of bribery being discussed; bribery has been described as prevalent throughout Romanian healthcare (Stan, 2015) but was not mentioned by Romanian participants.

## Conclusion

This study focused on migrant families’ experiences of primary healthcare and health promotion, subjects which have been insufficiently explored in research. The study breaks new ground in exploring parents’ views of child health promotion post-migration to the UK. The UK health system challenges migrant parents’ expectations of health professional/service user relationships and the power relations between parent, child and professionals. To improve the health of migrant children, primary care and health promotion practitioners require knowledge and understanding of the individual experiences of migrant families in order to develop their own skills in facilitating service users’ adjustment to a new model of healthcare.
